# Telehealth as an Element of Home and Community-Based Services in a Pandemic: An Intrinsic Case Study in Two Rural Areas

**DOI:** 10.1093/geroni/igab046.2742

**Published:** 2021-12-17

**Authors:** Nancy Karlin, Joyce Weil

**Affiliations:** 1 University of Northern Colorado, Greeley, Colorado, United States; 2 University of Northern Colorado, University of Northern Colorado, Colorado, United States

## Abstract

COVID-19 has changed the face of health care delivery. Using technology as a way to ensure Home and Community-Based Services (HCBS) as an option for older adults in rural areas is of increasing interest as a result of the pandemic. Literature suggests older adults do not adopt telehealth and/or medicine practices due to barriers (e.g., Internet and computer availability) and do not use telemedicine as a form of communication with medical staff. However, the combination of needing health care during the pandemic and having federal coverage via Medicare for telehealth virtual visit. Still studies suggest older adults may lack the necessary information about how to adopt telehealth and telemedicine and that they do not see their benefits. Additionally, the cost of technology, limited Internet access and rural connectivity issues persist. This study evaluates the potential for telehealth/medicine use in rural communities through two case studies of rural older persons in the Eastern Plains of Colorado and rural Western Nebraska. Results indicate, for older persons responding to the telehealth/medicine questions, there is support for its potential use with some using teleconferencing, health portals, along with the expectation that telehealth/medicine would be part of new health care systems. Resistance was met by some older adults in the Colorado sample who preferred face-to-face contact alongside other concerns about potential usage barriers such as the lack of Internet services or consistent connectivity. These participants indicated a lack of awareness in finding out how to access this form of medical support.

